# OA15.01. Use of self-care habits among graduates of the HEART program and association with physician burnout

**DOI:** 10.1186/1472-6882-12-S1-O58

**Published:** 2012-06-12

**Authors:** M Dossett, W Kohatsu, W Nunley, D Mehta, R Davis, R Phillips, G Yeh

**Affiliations:** 1 Beth Israel Deaconess Medical Center, Boston, USA; 2 Santa Rosa Family Medicine Residency, Santa Rosa, USA; 3 CareOregon, Portland, USA; 4 Massachusetts General Hospital, Boston, USA

## Purpose

Physician burnout is associated with decreased well-being, loss of empathy, and reduced quality of patient care. Despite high rates of burnout among physician trainees, few studies have explored strategies to address this problem. We examined the skills and habits associated with decreased burnout among physicians who participated in the Humanistic Elective in alternative medicine, Activism, and Reflective Transformation (HEART), an innovative fourth year medical student elective teaching humanism, physician self-care, and integrative medicine.

## Methods

We conducted a cross-sectional survey of HEART alumni from 2002-2009, collecting information on demographics, medical training, self-care habits, burnout (Maslach Burnout Inventory), mindfulness (Baer Mindfulness Questionnaire) and experience with the elective, both quantitatively and qualitatively. We used descriptive statistics to characterize the sample, grounded theory to inform the qualitative analyses, and multivariable linear regression to determine which self-care habits were associated with reduced burnout.

## Results

Of 168 eligible alumni, 122 (73%), completed the survey. The majority were female (71%), age ≤35 (78%), and trained in primary care specialties (67%). Forty-two percent were residents in training. The majority of respondents highly agreed that the elective helped them better cope with stress during residency training (80%), taught them self-care skills (75%), and improved their ability to empathize and connect with patients (71%). Qualitative analysis of the personal and professional impact of the elective identified twelve common themes with self-care, self-discovery, and collegial development/community most frequently cited. Practice of self-care habits was common in this cohort. After adjusting for age, gender, and stage of training the self-care habits most associated with reduced burnout were reflection (beta=3.7, p=0.02), social support (beta=8.1, p=0.003), and mindfulness (1.5 points/std dev change, p=0.02).

## Conclusion

The HEART curriculum promotes humanism and self-care skills that may protect against physician burnout. Incorporating aspects of this curriculum more broadly into medical education may improve trainee well-being and patient care.

